# Bio‐inspired adhesive hydrogel for biomedicine—principles and design strategies

**DOI:** 10.1002/SMMD.20220024

**Published:** 2022-12-25

**Authors:** Wenzhao Li, Xinyuan Yang, Puxiang Lai, Luoran Shang

**Affiliations:** ^1^ Zhongshan‐Xuhui Hospital and the Shanghai Key Laboratory of Medical Epigenetics the International Co‐laboratory of Medical Epigenetics and Metabolism (Ministry of Science and Technology) Institutes of Biomedical Sciences Fudan University Shanghai China; ^2^ Department of Biomedical Engineering The Hong Kong Polytechnic University Hong Kong China; ^3^ The Hong Kong Polytechnic University Shenzhen Research Institute Shenzhen China

**Keywords:** adhesion, bio‐inspired, biomaterial, biomedical, hydrogel, interface

## Abstract

The adhesiveness of hydrogels is urgently required in various biomedical applications such as medical patches, tissue sealants, and flexible electronic devices. However, biological tissues are often wet, soft, movable, and easily damaged. These features pose difficulties for the construction of adhesive hydrogels for medical use. In nature, organisms adhere to unique strategies, such as reversible sucker adhesion in octopuses and nontoxic and firm catechol chemistry in mussels, which provide many inspirations for medical hydrogels to overcome the above challenges. In this review, we systematically classify bioadhesion strategies into structure‐related and molecular‐related ones, which cover almost all known bioadhesion paradigms. We outline the principles of these strategies and summarize the corresponding designs of medical adhesive hydrogels inspired by them. Finally, conclusions and perspectives concerning the development of this field are provided. For the booming bio‐inspired adhesive hydrogels, this review aims to summarize and analyze the various existing theories and provide systematic guidance for future research from an innovative perspective.

## Graphical abstract

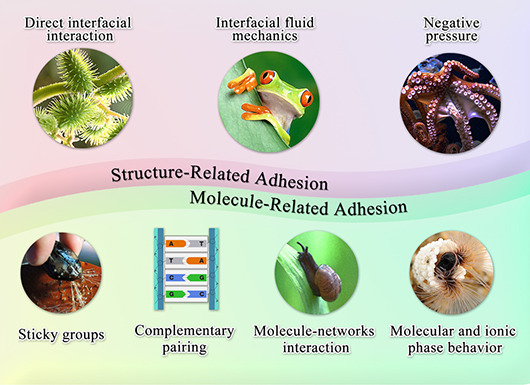

General classification of biological adhesion strategies. Specifically, the corresponding examples are the hook‐like structures of cocklebur, the footpads of tree frogs, octopus suckers, catechol groups in mussel adhesives, base‐complementary pairing, snail mucus, and sandcastle worm secretions with phase behavior.

1

**Key points**

- The bioadhesion mechanism of bio‐inspired adhesion hydrogels are categorized systematically into structure‐related and molecule‐related types.
- The corresponding design criteria of bioadhesive hydrogels inspired by these principles are introduced.
- This review also focuses on the nascent research results and undetermined viewpoints in this field and attempts to propose more innovative perspectives.

## 2 INTRODUCTION

Hydrogel is a kind of three‐dimensional polymer network with high water content. It holds great promise in the biomedical field due to the tissue‐like soft and wet matrix, diverse and tunable physicochemical properties, and potentials for functionalization.^1^, ^2^, ^3^, ^4^ Common applications include medical patches, tissue sealants, drug carriers, and flexible electronic devices.^5^, ^6^, ^7^, ^8^, ^9^, ^10^, ^11^ Generally, tissue adhesion is an important consideration for hydrogels to be applied in biomedical fields. For example, medical dressings and patches need to be attached to the tissue surface for protection and support, occlusive agents need to stick to the tissue under certain stress, and microgel carriers are expected to adhere to and reside in a specific location.^12^, ^13^, ^14^, ^15^, ^16^, ^17^, ^18^ However, bioadhesive hydrogels need to deal with complex and changeable physiological environments and specific application demands. First, the hydrogel‐tissue interface tends to be wet. The hydration layer formed by water molecules at the interface will impact the molecular‐level forces; the macroscopic interfacial water also reduces the effective area of the adhesion surface.^19^, ^20^, ^21^ Second, the tissue surface is elastic and soft with various movements. Adhesion should not only be strong but also stretchable, without rigid interfaces or detachment during motion.^22^, ^23^, ^24^, ^25^, ^26^ In addition, bioadhesive hydrogels should have good biocompatibility and avoid causing chemical toxicity or mechanical damage to the tissue.^27^, ^28^, ^29^, ^30^

The excellent and unique adhesion ability of various organisms in nature provides inspiration for solving these challenges. Part of biological adhesion relies on unique structures, such as the suction cup of octopus,^31^, ^32^, ^33^, ^34^, ^35^ the disc of clingfish,^36^, ^37^ the barb‐like or burred structure of hookworm and cocklebur,^38^, ^39^ the hierarchically structured feet of gecko,^40^, ^41^, ^42^, ^43^ and the patterns of tree frog toes.^44^, ^45^, ^46^ Another part of the adhesion ability relies on specific molecular‐level non‐structure‐related forces. This can be originated from, for example, sticky components of mussel adhesives,^47^, ^48^, ^49^, ^50^ entanglement of macromolecules in mucus,^51^, ^52^ the complementary pairing of receptor‐ligand,^53^, ^54^ and phase behaviors such as coacervation, solidification, and biomineralization of sandcastle worms.^21^, ^55^, ^56^, ^57^, ^58^, ^59^, ^60^, ^61^ Intriguingly, these natural strategies have recently been well implemented in the design of hydrogels to achieve bioadhesion. The resultant hydrogels have found widespread applications in biomedicine, including patches with interlocking microneedles,^62^, ^63^, ^64^, ^65^ suction cups,^66^, ^67^, ^68^, ^69^ and hydrogels grafted with sticky groups,^70^, ^71^, ^72^, ^73^ etc.

In this review, we summarize the principles and design strategies of bio‐inspired adhesive hydrogels in the context of biomedicine. We systematically categorize the bioadhesion mechanism into structure‐related and molecule‐related types, each containing several specific principles, shown as Graphical Abstract. Besides, the corresponding design criteria of bioadhesive hydrogels inspired by these principles are introduced. Finally, we discuss the current achievements, challenges, and future development of this field. With this, we aim to provide a concise yet systematic description and critical thinking of bio‐inspired hydrogel for biomedicine. This review also focuses on nascent research results and undetermined viewpoints in this field and attempts to propose more innovative perspectives. We believe that this paper would trigger more discussions about biological adhesion principles and biomimetic material design strategies, ultimately promoting the development of the field.

## 3 STRUCTURE‐RELATED ADHESION

**FIGURE 1:**
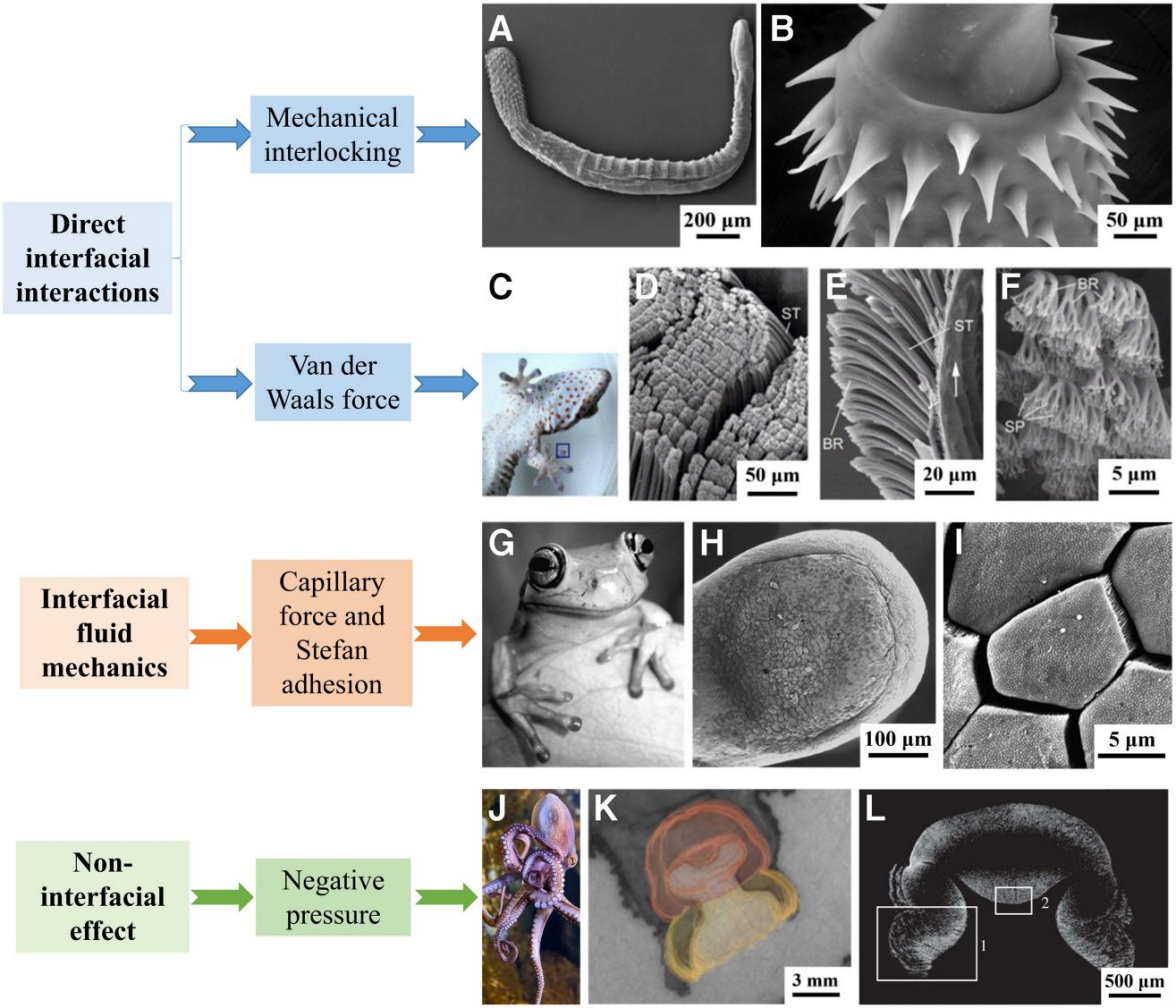
Structure‐related adhesion mechanisms in nature organisms. (A) SEM image of habitus of *Serrasentis sagittifer*. Reproduced under terms of the CC‐BY license.^75^ Copyright 2011, The Authors, published by PLOS. (B) SEM image of clear anterior trunk spines of proboscis. Reproduced under terms of the CC‐BY license.^76^ Copyright 2014, The Authors, published by EDP Sciences. (C) Image of a gecko; the framed area is its toe. Reproduced with permission.^77^ Copyright 2005, Elsevier. (D–F) SEM images of the (D) arranged setae (ST) structures, (E) rich branches (BR) of setae, and (F) distal spatulae (SP) of branches. Reproduced with permission.^77^ Copyright 2005, Elsevier. (G) Image of a tree frog (*Litoria Caerulea*). Reproduced with permission.^74^ Copyright 2014, Taylor & Francis. (H, I) SEM images of (H) a toe pad of a tree frog and (I) epidermis on toe pad that form a hexagonal pattern. Reproduced with permission.^74^ Copyright 2014, Taylor & Francis. (J) Image of an octopus attached to a glass surface with funnel‐shaped suckers. Reproduced with permission.^34^ Copyright 2002, Oxford University Press. (K) Histological image and 3D reconstruction of sucker structures. Reproduced under terms of the CC‐BY license.^33^ Copyright 2013, The Authors, published by PLOS. (L) Microcomputed tomography image of an octopus's suckers. The acetabular wall is shown as one and the protuberance is shown as 2. Reproduced under terms of the CC‐BY license.^78^ Copyright 2013, The Authors, published by the Royal Society. Scale bar is 200 μm in (A), 50 μm in (B) and (D), 20 μm in (E), 5 μm in (F) and (I), 100 μm in (H), 3 mm in (K), and 500 μm in (L).

In nature, various structure‐related strategies have evolved on the surface of biological adhesion organs.^74^ According to whether the adhesion comes from the interface, and whether it is related to the fluid medium, we divide the common ordered structure‐related bioadhesion strategies into three main categories: direct interface interaction‐related, interface fluid mechanics‐related and negative pressure‐related. Among them, the direct interfacial interaction mainly refers to the mechanical interlocking (Figure 1A,B) and van der Waals forces (Figure 1C–F) enhanced by the ordered structure.^79^, ^80^, ^81^, ^82^ The second type, the adhesion force originated from interface fluid mechanics is mainly reflected in the considerable capillary force or Stefan adhesion brought by the ordered structure (Figure 1G–I).^20^, ^83^, ^84^ Since excessive interfacial water often has a negative effect on adhesion, interface fluid mechanics‐related strategies may also include those structures favorable for water drainage.^9^, ^21^ The third type, negative pressure‐dependent adhesion is often based on a suction cup‐like structure (Figure 1J–L). Interestingly, it does not originate from the interface where adhesion occurs. The adhesion force is essentially determined by the pressure difference inside and outside a negative pressure chamber. The force occurs at the interface between the negative pressure structure and the external fluid and is transferred to the adhesive interface.^85^ In this section, we introduce classical structure‐related bioadhesive mechanisms and the strategies for the construction of corresponding bio‐inspired hydrogels.

### 3.1 Direct interfacial interaction

As mentioned above, direct interfacial interaction‐related adhesion structures mainly correspond to two effects—mechanical interlocking and van der Waals forces. Examples of mechanical interlocking in nature organisms include mayfly larvae, thorny‐headed worms (Acanthocephala) (Figure 1A,B), cockleburs, etc.,^38^, ^39^, ^79^, ^86^ which tend to have barb‐like or similar structures. Biological organs with such structures can plunge into the matrix for a firm anchoring. Especially in some species, this structure can have dynamic responsiveness. For example, *Pomphorhynchus laevis* can expand a bulb of proboscis after the insertion of the thorn‐like structure. When the mechanically interlocked structure expands after insertion, it exerts a greater force with the matrix like an expansion bolt, further strengthening the anchorage.^87^

**FIGURE 2:**
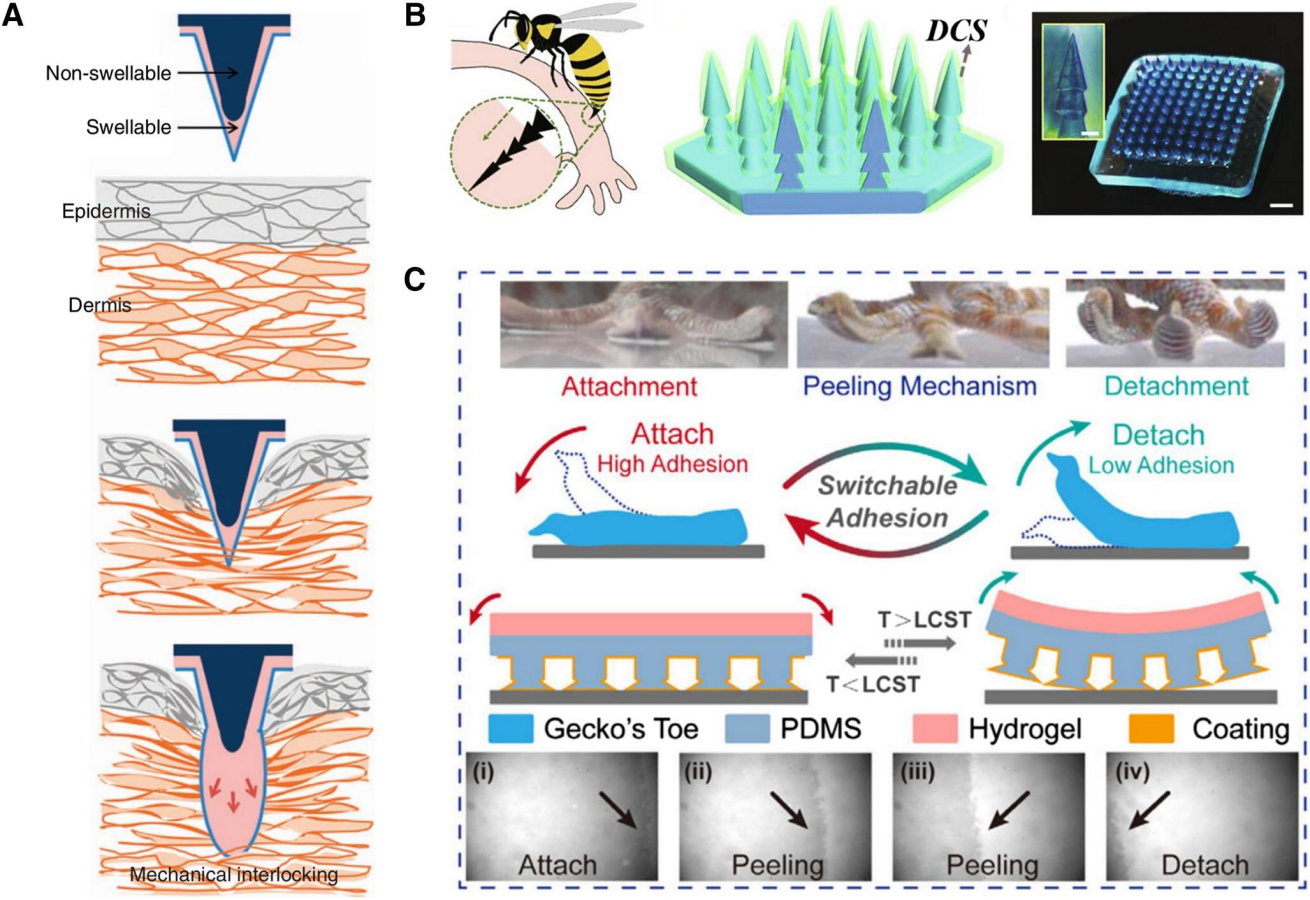
Bio‐inspired artificial hydrogel with direct interface interaction‐related structures. (A) Mechanical interlocking of a double‐layer microneedle via hydrogel swelling. Reproduced with permission.^63^ Copyright 2013, Springer Nature. (B) Sting‐inspired hierarchical microneedle array with mechanical interlocking‐mediated adhesion. Reproduced with permission.^62^ Copyright 2021, Elsevier. (C) Gecko‐inspired hydrogel patch. The thermally responsive hydrogel back sheet realizes controllable attachment and detachment. Reproduced with permission.^88^ Copyright 2021, American Chemical Society.

The mechanical interlocking strategy has inspired the construction of many adhesive hydrogels for medical use, mainly microneedle patches. Yang et al. reported a swellable double‐layer microneedle adhesive (Figure 2A).^63^ Before contacting with water, the smooth needles can be easily inserted into the tissue in a dry and hard state. By absorbing water in tissues, a rapid increase in the cross‐sectional area occurs, achieving local tissue deformation and subsequent interlocking, and thus providing adhesion. As the hydrogel swells, the soft microneedle tip can be removed without significantly damaging the tissue or causing the microneedle to break during rigid removal. Besides, Zhang et al. fabricated a mechanically interlocking hydrogel microneedle patch with a multi‐layered structure by step‐by‐step mold replication (Figure 2B).^62^ The interlocking structure can make the microneedle tightly anchored to the surface of the tissue.

Another type of direct interfacial interaction is the substantial Van der Waals force provided by hairy structures of hierarchical branches of some reptiles and insects.^89^ Among them, the most widely studied gecko toe has a complex multi‐level structure from macroscale to nanoscale, including lamellae, setae, branches, and spatulae (Figure 1C–F).^43^ Such hierarchy in structure enables the spatulae to match the roughness of a surface to be adhered, increasing the effective contact area and resulting in considerable van der Waals forces. In addition, geckos can effectively adhere and peel by changing the angle between the toe and the surface to be adhered.^80^, ^82^ According to the latest research, the contribution of forces such as acid‐base interactions in gecko adhesion has also been proposed.^90^ However, van der Waals forces are still considered to be the main source of adhesion. Also interestingly, it remains a question whether the presence of interfacial water has positive or negative effects on gecko adhesion.^91^, ^92^, ^93^, ^94^

For gecko‐inspired hydrogels, the adhesive structures are abstracted as arrays of columns of various types, which are usually prepared by template methods. Interestingly, an “array + coating” design paradigm has been implemented to construct patches that combine the high van der Waals forces of gecko‐inspired arrays with other adhesive properties of hydrogel surfaces.^95^ Mahdavi et al. utilized photolithography and reactive ion etching to prepare silicon templates with arrays of concave pillars of various shapes. A prepolymer is then perfused and cross‐linked, resulting in a gecko‐inspired column array. The array surface was further modified with oxidized dextran (DXT) coating for covalent adhesion to tissues. Zhou et al. improved this “array + coating” paradigm.^96^ A thermally responsive adhesive macromolecule p(DMA‐co‐MEA‐co‐NIPAm) was coated with an array of mushroom‐shaped pillars with enlarged heads. The hydrophilic–hydrophobic transition of this coating is controlled by temperature. Excessive interfacial water can lead to failures of such as van der Waals forces on the surface of the gecko array. Similarly, the artificial array adhesion behavior is controlled by temperature‐controlled water contact angle transition. Zhang et al. imitated the peeling behavior of geckos on previous research basis.^88^ The controllable peeling of the patch was achieved by integrating a thermally responsive bending hydrogel on the back sheet of the patch (Figure 2C).

### 3.2 Interfacial fluid mechanics

In the presence of interfacial fluids, a class of bioadhesive surfaces relying on capillary force and Stefan adhesion is summarized. Tree frogs, clingfish, and flies are typical examples, whose adhesive organs often have special micro–nano array structures and concomitant mucous glands.^37^, ^44^, ^45^, ^46^, ^97^, ^98^, ^99^ The footpads of tree frogs have a hierarchical structure (Figure 1G–I). Epithelial cells present an array of polygon prisms, with the prisms spaced from each other forming channels. Through the channels, mucus may diffuse and excess interfacial water can be drained. Similarly, the feet of flies have brush‐like bristles, whose tip secretes a viscous liquid when contacted.^98^, ^100^, ^101^ Such liquids on the interfaces form numerous liquid bridges at the interface, thus providing the adhesion force that can be attributed to capillary force and Stefan adhesion.^45^, ^46^ The capillary force is formed by the attraction of the liquid to the surface and the cohesion of the liquid. Theoretical calculations reveal that when the total liquid volume remains unchanged, the capillary force increases significantly with the increase of the number of liquid bridges, which also explains part of the reason for the huge capillary force of the multilayer structure.^102^ Stefan adhesion occurs when two surfaces with fluid at the interface are separated from each other. It is a type of adhesion that is positively related to the viscosity of the liquid, the speed of interfacial separation, etc. Therefore, the highly viscous mucus has provided a considerable contribution to Stefan adhesion.^20^, ^103^

**FIGURE 3:**
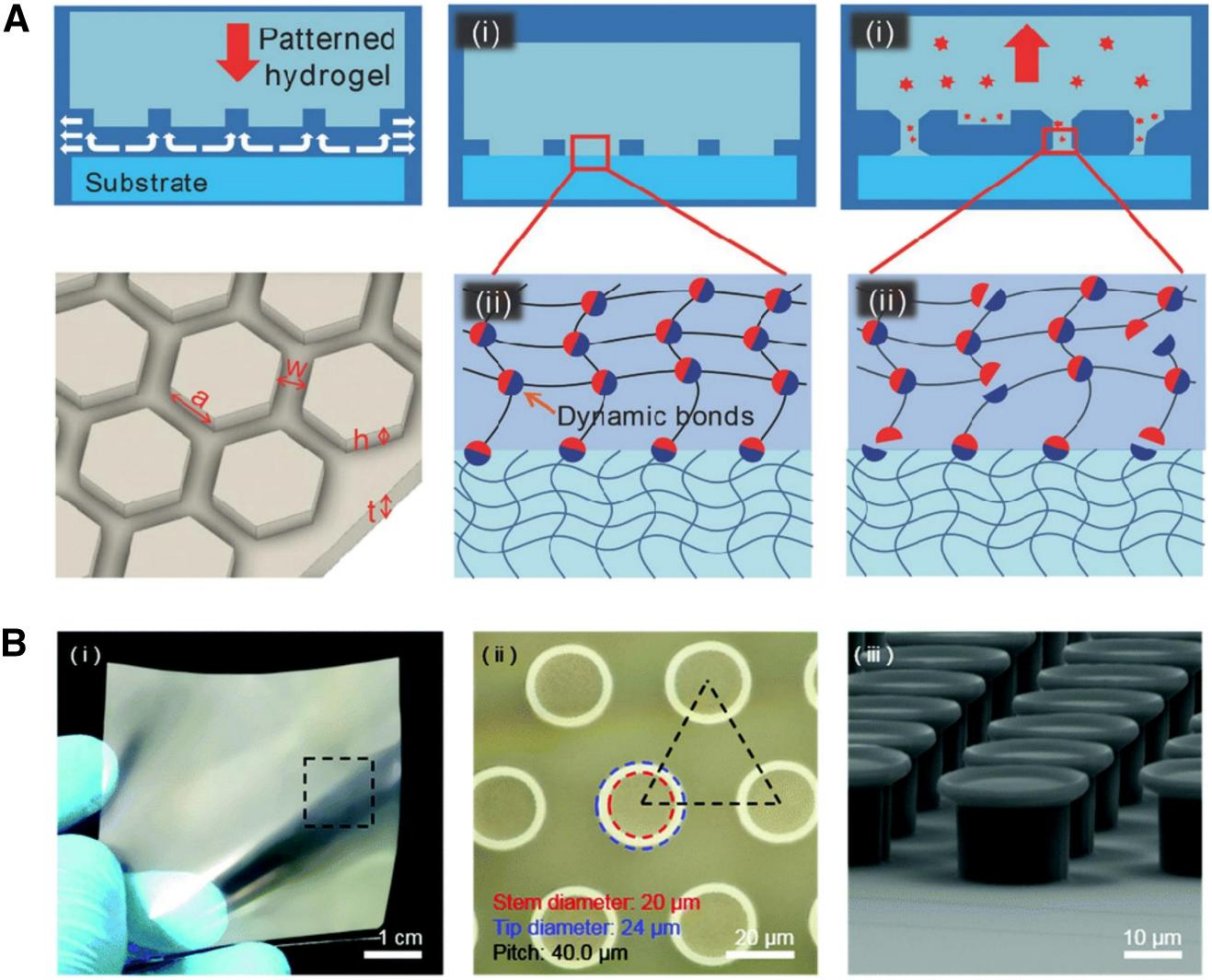
Bio‐inspired adhesive hydrogel with interfacial fluid mechanics‐related structures. (A) Clingfish disc‐inspired hydrogel with hexagonal arrays. When the hydrogel is in contact with the substrate, the groove drains rapidly. The dynamic bonds of the hydrogel bond to the matrix and dissipate energy when breaking. The hexagonal facets are separate from each other, which prevents crack propagation. Reproduced with permission.^104^ Copyright 2018, Wiley‐VCH. (B) Bio‐inspired hydrogel patch. Microarrays of hydrogels absorb water and maximize capillary adhesion forces. Reproduced with permission.^107^ Copyright 2018, The Royal Society of Chemistry.

The biomimicry of such structures is mainly reflected in two aspects, the drainage effect of a groove pattern on the interface water^104^, ^105^ and the capillary force and Stefan adhesion generated by an array in the presence of viscous glue.^106^, ^107^, ^108^ Rao et al. used the template method to construct a class of hexagonal facet arrays with interconnecting grooves (Figure 3A).^104^ When the hydrogel contacts with an adhered surface, the interfacial water is drained from such grooves, and the interface forms a good contact. The matrix of the hydrogel was designed based on dynamic bonds. The high energy dissipation when broken endowed the hydrogel with good adhesion and toughness. In addition, the segmented hexagonal facet. also prevented the continuous propagation of cracks at the interface. Bo et al. constructed similar interconnecting grooved hexagonal facets using thermally triggered shape memory hydrogels.^105^ Under thermal triggering, the hexagonal column structure of the hydrogel was transformed into a flat structure that was not conducive to adhesion, and accordingly, the adhesion strength decreased to 15.4% of the initial value. Meng et al.'s work is not limited to the patterned surface itself but focuses more on the interaction between the pattern and the fluid at the interface.^106^ The effects of different temperatures, heights of hexagonal columns, and viscosity and surface tension of the interfacial fluids on the adhesion behavior were studied. Finally, the optimal conditions for realizing low separation distance, high adhesion, and high friction were obtained. Yi et al. adopted the principle that hydrogels coupling with a microarray structure could generate huge capillary and van der Waals forces to produce reversible and strong adhesion on dry, wet, and underwater surfaces (Figure 3B).^107^

### 3.3 Negative pressure

Negative pressure is a non‐interfacial effect. Many species maintain adhesion at the interface through negative pressure. The typical examples include the suction cup of octopus, the foot of snails, and the disc of clingfish.^31^, ^32^, ^33^, ^34^, ^35^, ^36^, ^37^, ^51^ Taking octopus as an example, the acetabulum cavity inside the suction cup forms negative pressure through muscle contraction and other methods, and the outer edge forms a seal (Figure 1J–L).^33^, ^34^, ^35^ Some micro/nanostructures common on the surface of the adhesive organs have the effect of consolidating the seal and enhancing the friction. Adhesion by suction often occurs at a wet interface, which is related to the maintenance of the negative pressure by water in the cavity and interface. On non‐wettable surfaces or other scenarios prone to cavitation, the fail of water in tension may result in the fail of negative pressure adhesion.^35^, ^85^, ^109^ Interesting recent studies have revealed that two chambers can be identified when the suction cup adheres to a substrate, including an acetabular chamber and an infundibular chamber. The trapped water in the infundibular chamber is also considered to contribute significantly to suction.^110^, ^111^, ^112^ The multilevel fine structure and muscle‐driven contraction patterns allow for strong adhesion at various interfaces while being reversibly detachable. This ensures that octopuses can both anchor themselves to rocks in turbulent currents and move by loosening their suction cups.^113^, ^114^, ^115^ The continuous exploration of the principle will lead to the emergence of more versatile microstructured medical hydrogels.

**FIGURE 4:**
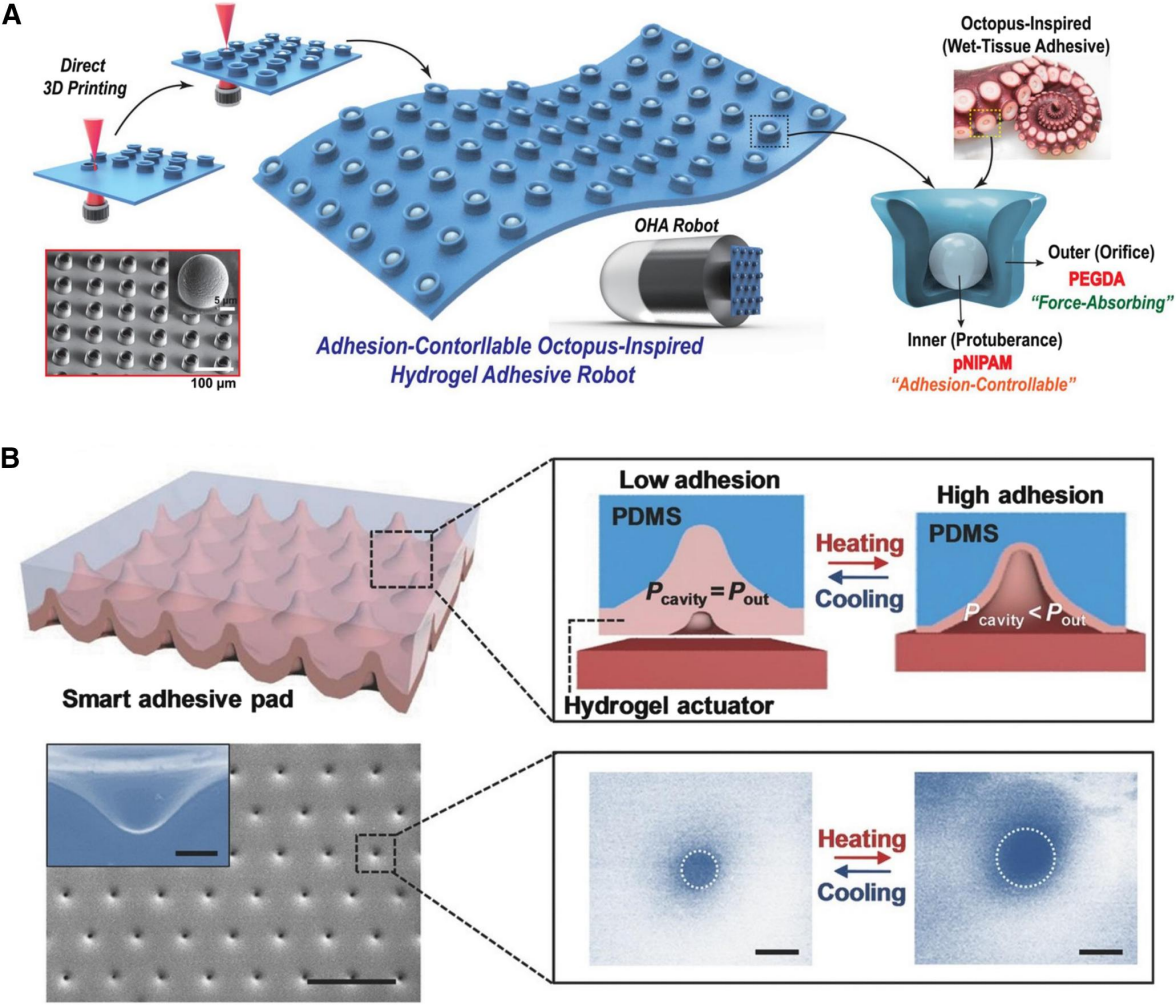
Bio‐inspired artificial hydrogel with suction cup‐like structures generating negative pressure for adhesion. (A) An octopus‐inspired hydrogel patch. The wall of the sucker is a PEGDA hydrogel and the internal protruding structure is composed of pNIPAM hydrogel. Reproduced with permission.^118^ Copyright 2022, The Authors, published by Wiley‐VCH. (B) Intelligent adhesive pad with suction cup‐inspired microcavity structure. The adhesion is temperature sensitive. Reproduced with permission.^117^ Copyright 2016, Wiley‐VCH.

The structures that produce negative pressure adhesion in nature have inspired hydrogels with various types of suction cups or cavity structures. The preparation methods involved include the template method,^66^, ^68^, ^116^, ^117^ 3D printing,^118^, ^119^ liquid or air trapping,^67^, ^112^, ^120^, ^121^ etc. Fu et al. used the lithography technology to construct a mold with suction cup structures, and finally fabricated a patch.^68^ Lee et al. developed an advanced 3D microprinting technique based on two‐photon polymerization to construct heterostructures (Figure 4A).^118^ The formed hydrogel suction cup was composed of PEGDA on the outer wall and pNIPAM spherical protrusions inside. pNIPAM is a class of thermosensitive hydrogels that can generate heat‐triggered shrinkage. Such temperature‐sensitive shrinkage of the spherical protrusions resulted in a change in the effective suction area, which enabled the patch to have a higher adhesion at 27°C relative to that at 45°C. Similarly, Ko and colleagues coated pNIPAM hydrogels on elastomeric microcavity pads made of PDMS to mimic the contraction behavior of octopus suckers (Figure 4B).^117^ The smart adhesive pad exhibited thermally controllable adhesion, which was high at 35°C and low at 22°C. Baik et al. developed a series of “air‐trapped” methods.^112^, ^120^ They used air bubbles to controllably form gas–liquid interfaces in an array of holes to prepare suction cups with concave columns with spherical protrusions inside.^112^ Cai et al. prepared suction cup structures based on an assembly of colloidal particles in droplet templates.^67^ The resulting sucker‐shaped microspheres containing self‐assembled nanoparticles (NPs) were obtained through the fast water extraction process in the droplets.

At the end of the discussion of the main bioadhesion structures, it is worth mentioning that in nature, especially underwater, the forces corresponding to each structure do not appear alone. For example, the adhesion of octopus sucker actually involves multiple mechanisms, including capillarity, friction, and negative pressure, while the contribution of the capillary force in gecko toes' adhesion behavior is still controversial.^34^, ^35^, ^82^, ^91^, ^111^ This review can only relate specific structures to their primary mediated effects. For bio‐inspired structure‐related artificial adhesive hydrogels, the design is often not only inspired by a single organism or limited to a single effect. Therefore, the strict imitation of nature is difficult and somewhat unnecessary in the application aspect. The soft and wet properties of hydrogels make it difficult to be shaped into precise micro–nano structures identical as that of the adhesive organs in natural organisms. However, the unique features of hydrogels such as swelling and temperature response also endow the artificial adhesion materials with more flexible design and more functional potentials, making it possible to surpass natural materials in terms of controllability and adhesion ability.

## 4 MOLECULE‐RELATED STRATEGIES

**FIGURE 5:**
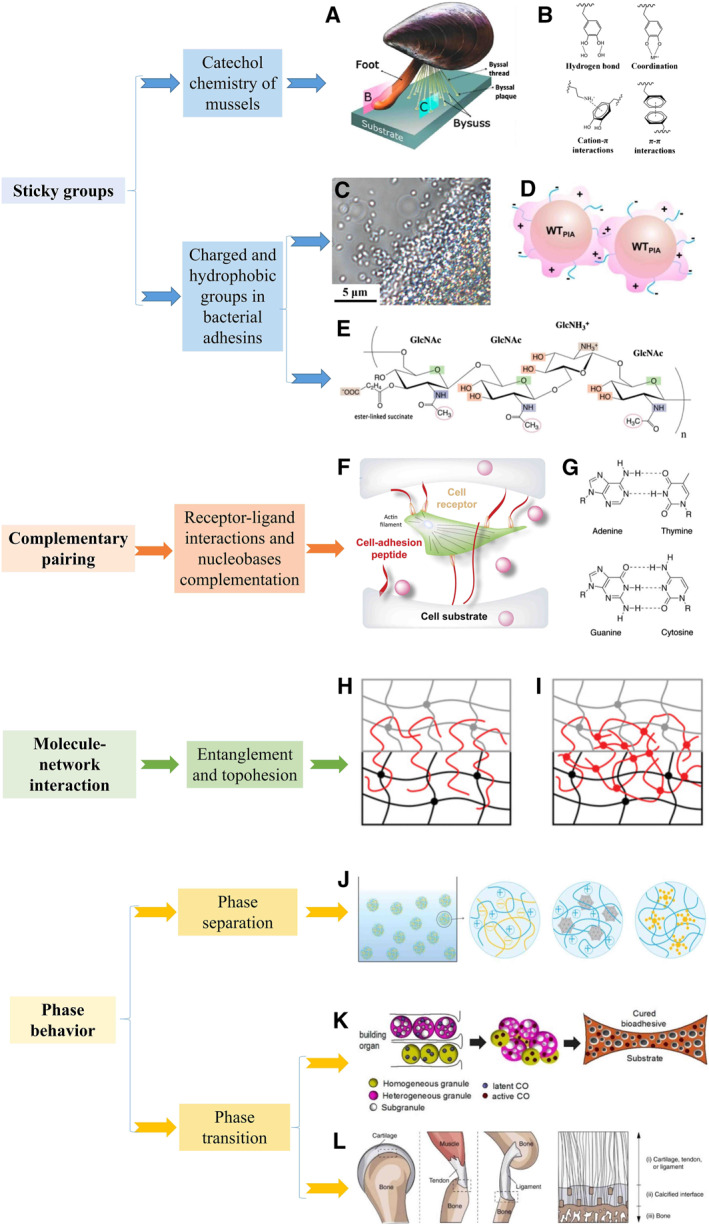
Molecule‐related adhesion strategies in nature, including their action forms, typical biological examples, and principles. (A) Mussel adhesion. Reproduced with permission.^47^ Copyright 2017, American Chemical Society. (B) Some common interaction paradigms of catechol chemistry. Reproduced with permission.^122^ Copyright 2021, Wiley‐VCH. (C) Microscope image of bacteria with adhesins. The scale bars are 5 μm. Reproduced with permission.^123^ Copyright 2016, American Chemical Society. (D) Scheme of bacteria with charged adhesins. Reproduced with permission.^123^ Copyright 2016, American Chemical Society. (E) Molecular structure of adhesin. Reproduced with permission.^124^ Copyright 2013, American Chemical Society. (F) Cell adhesion on a surface mediated by receptor–ligand interaction.^125^ Copyright 2018, Elsevier. (G) Nucleobase complementary pairing. (H, I) Entanglement and topohesion. Reproduced with permission.^26^ Copyright 2020, Wiley‐VCH. (J) Liquid–liquid phase separation by coacervation. Reproduced with permission.^21^ Copyright 2021, Wiley‐VCH. (K) Sandcastle worm adhesives' phase transitions. Reproduced with permission.^126^ Copyright 2013, American Chemical Society. (L) The calcified interface between various tissues and bone. Reproduced under terms of the CC‐BY license.^25^ Copyright 2020, The Authors, published by Springer Nature.

According to the spatial scales and characteristics of molecule‐related bioadhesion strategies in nature, we classify them into four categories: sticky groups, complementary pairing, molecule–network interaction, and phase behavior. Generally speaking, the spatial scale varies from small to large, sticky groups and complementary pairing can occur within a few molecules, while molecule–network interaction requires macromolecules and their networks, and phase behavior can even be observed at the micrometer scale.^21^, ^26^, ^47^, ^55^ Sticky groups are commonly found in catechol chemistry of mussels (Figure 5A,B) and various charged, hydrophobic groups, for example, in adhesins of bacterials (Figure 5C–E).^70^, ^71^, ^72^, ^73^, ^127^, ^128^ Such adhesion is often non‐specific, which means that materials with similar groups can adhere to a wide range of surfaces. Molecular complementary pairing‐derived adhesion is supposed to be more specific. In organisms, it is mainly manifested as receptor–ligand interactions (Figure 5F) and complementary pairing of nucleobases (Figure 5G).^125^, ^129^, ^130^ Hydrogels inspired by this tend to adhere to specific surfaces. In the molecule–network interaction scale, entanglement (Figure 5H) and topological adhesion (topohesion) (Figure 5I) are discussed together. These processes involve molecular chains and the interactions with the adhered network.^56^, ^57^, ^59^, ^131^, ^132^ Phase behaviors related to adhesion include phase separation, mainly coacervation (Figure 5J), and phase transitions, including solidification and mineralization (Figure 5K,L).^55^, ^57^, ^59^, ^60^, ^61^, ^133^, ^134^, ^135^ Similar to the previous chapter, various biological molecular‐related adhesion principles and corresponding artificial adhesion strategies of hydrogels are discussed.

### 4.1 Molecules with sticky groups

Mussels, sandcastle worms, and barnacles adhere by secreting molecules with sticky groups.^47^, ^48^, ^49^, ^50^, ^55^, ^136^, ^137^ Taking the most widely studied mussels for example (Figure 5A), they secrete a liquid‐state protein‐based glue called mussel foot protein (mfp). Further investigation reveals that the catechol group in 3,4‐dihydroxyphenyl‐l‐alanine is an important sticky group.^50^ The diverse interaction forms of catechol groups not only provide adhesion for the adhesive interface but also provide cohesion for the adhesive itself. These two effects together contribute to the adhesion. Specifically, the catechol groups can form noncovalent interactions, such as bidentate coordination, hydrogen bonds, hydrophobic interaction, π‐π, etc., with different substrate surfaces (Figure 5B).^138^, ^139^, ^140^, ^141^ Besides, the quinone structure after being oxidized can covalently react with –NH2, –SH, and other groups through Schiff base, Michael addition, or react with itself by dopa quinone coupling.^139^, ^142^ Of course, the actual mussel adhesion in nature involves more complex factors, including controlled changes in pH, redox, etc.,^143^, ^144^ far from just the action of catechol chemistry. The coacervation and solidification of mussel adhesives are also as important and will be specifically described in Section 3.4.

**FIGURE 6:**
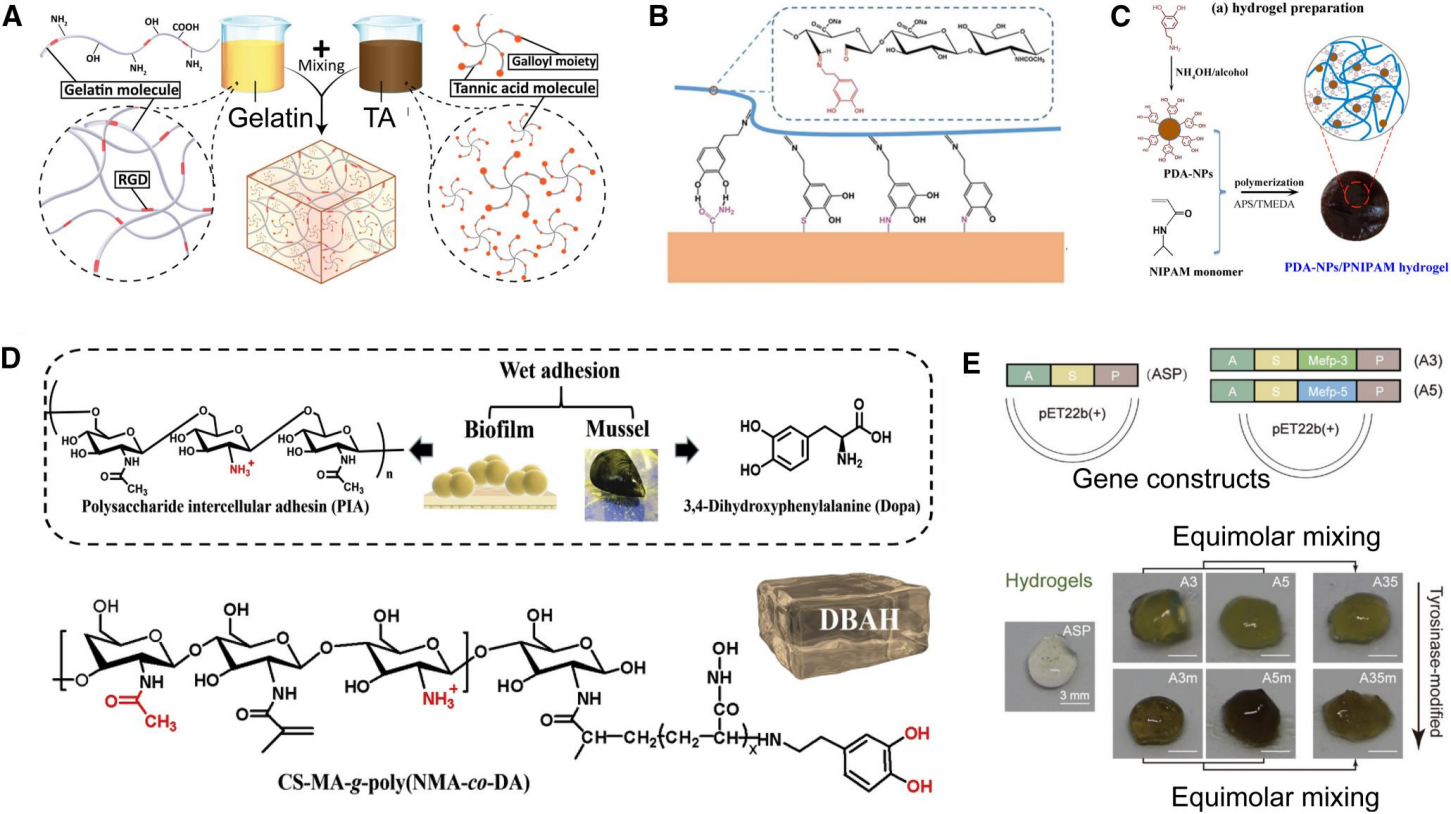
Bio‐inspired artificial hydrogels with sticky groups. (A) Adhesive hydrogel prepared by direct mixing of gelatin and TA. Reproduced with permission.^145^ Copyright 2021, Wiley‐VCH. (B) Hydrogel prepared by covalent grafting of dopamine on hyaluronic acid through Schiff base reaction. Reproduced with permission.^151^ Copyright 2020, American Chemical Society. (C) PDA‐NPs/PNIPAM hydrogel with simultaneous adhesion and temperature sensitivity. Reproduced with permission.^153^ Copyright 2016, American Chemical Society. (D) Dual bacterial biofilm‐ and mussel‐inspired hydrogel. Reproduced with permission.^157^ Copyright 2020, The Authors, published by Elsevier. (E) Adhesive hydrogel combining mussel adhesion mechanism and genetic engineering technology. Reproduced with permission.^158^ Copyright 2022, Wiley‐VCH.

Small molecules containing catechol‐like groups such as dopamine (DA) and tannic acid (TA) are widely introduced in artificial mussel‐inspired adhesive hydrogels. Due to the wide range of chemical reaction forms, there are a variety of methods for introducing these groups. Catechol groups are typically introduced into hydrogels by direct mixing of these small molecules,^145^, ^146^, ^147^ covalently grafting on hydrogel networks,^70^, ^71^, ^72^, ^148^, ^149^, ^150^, ^151^, ^152^ and the use of nanomaterials.^73^, ^153^, ^154^, ^155^, ^156^ Doping and soaking are the easiest ways to non‐covalently introduce monomeric molecules. For example, Ahmadian et al. added TA directly to a gelatin solution. The hydroxyl groups of TA are hydrogen donors, while the amino and carboxyl groups of gelatin are hydrogen acceptors. The two compositions formed abundant hydrogen bonds and then physically cross‐linked to form a non‐toxic Gelatin‐TA hydrogel (Figure 6A).^145^ Chen et al. developed a method of polymerization lyophilization conjugation.^146^ PEGDA hydrogels were first prepared and lyophilized and then soaked in a TA solution. Due to the high flexibility of the PEGDA molecule and its high affinity to tannins, the gel showed good mechanical properties and underwater adhesion.

Covalent graft of DA can be achieved via the reaction of the amino group of DA with the carboxyl group in the side chain of macromolecules.^70^, ^71^ Schiff base or Michael addition can occur between the amino group or sulfhydryl group of other molecules and DA and TA, or between the amino group of DA and other unsaturated bonds.^150^, ^151^, ^152^ For example, Zhou et al. oxidized hyaluronic acid (HA) to generate aldehyde groups. Then the aldehyde group reacted with the amino group of DA to obtain DA‐modified HA. The catechol in the DAHA molecule can be cross‐linked by NaIO_4_, which enabled rapid formation of hydrogels with strong adhesion (Figure 6B).^151^

In addition, DA, TA, and other molecules can be combined with various inorganic non‐metallic and organic compounds or self‐polymerized to form nanomaterials. Doping of such nanomaterials will also enhance the hydrogel adhesion. Han et al. synthesized PDA nanoparticles by oxidative self‐polymerization of DA. The PDA nanoparticles were then directly incorporated into a PNIPAm pregel and cured. The obtained hydrogel had good cell‐tissue adhesion ability (Figure 6C).^153^ Feng et al. obtained PPy‐PDA nanoparticles by copolymerization of pyrrole (Py) and DA. The nanoparticles were then incorporated into PNIPAm hydrogels to obtain NIR‐tunable adhesion.^154^ Han et al. inserted DA into clay nanosheets in order to protect them from oxidation. Acrylamide monomers were further incorporated to form a composite hydrogel that exhibited broad, durable, and reproducible adhesion.^155^ Jia et al. used silver ions and TA to obtain TA‐Ag nanozymes via in situ reduction. The phenolic hydroxyl group of TA promoted its uniform incorporation into a polyacrylic acid hydrogel. The nanozyme in the hydrogel network maintained the balance of dynamic redox between phenol and quinone, thereby ensuring the stability of the hydrogel adhesion.^156^ It is worth mentioning that catechol chemistry can be incorporated not only in a homogeneous way but also heterogeneously through surface coating, which also provides more ideas for the construction of adhesive hydrogel materials.^159^, ^160^

Nonspecific sticky groups of microorganisms are also worth investigating. In order to achieve permanent adhesion to non‐biological surfaces under various complex conditions, various microorganisms have developed a series of universal non‐specific adhesins (Figure 5C–E). Many adhesins have adhesive abilities derived from sticky groups.^161^, ^162^, ^163^, ^164^ Several species of marine bacteria such as *Caulobacterale* have charged molecules on their holdfast that allow them to attach to surfaces.^161^ Increasing the expression of the polysaccharide deacetylase has demonstrated an adhesion enhancement effect.^162^, ^163^ Such nonspecific adhesin‐mediated adhesion has also inspired biomimetic design.^157^, ^165^ Inspired by the positively charged polysaccharide intercellular adhesin (PIA) in biofilms, Han et al. grafted chitosan to have similar structure and composition with natural PIA as a source of adhesion (Figure 6D).^157^ In addition, synthetic biology using engineered microorganisms can also help to construct hydrogels with adhesive groups.^158^, ^166^, ^167^, ^168^ Jiang et al. adopted a modular genetic strategy to design recombinant protein hydrogels incorporating Mefp‐3 and Mefp‐5 from mussels (Figure 6E).^158^ The final obtained protein‐based hydrogel had good adhesive strength. Another interesting example is the combination of the adhesion domains of mussel Mefp with the abilities of environmental perception, migration, and adhesin secretion of microorganisms by which a multifunctional adhesive “living glue” was prepared.^167^

### 4.2 Molecular complementary pairing

There are a lot of specific molecular complementary pairing phenomena in organisms such as base complementary pairing and receptor‐ligand interaction. These interactions can also contribute to bioadhesion and inspire material design. The mechanism of specific adhesion of cells is particularly instructive in the design of biomaterials. The specific adhesion of cells to cells or substrates is mediated by multiple receptor families, such as immunoglobulin superfamily, cadherins, and the widely studied integrins (Figure 5F).^125^, ^129^, ^169^, ^170^ A representative example is the interaction between integrin and short peptide Arg‐Gly‐Asp (RGD).^169^, ^171^, ^172^ RGD groups presenting on the surface of a matrix have been proved to promote cell adhesion. Cadherins are another well‐known class of cell adhesion molecules that mediate mechanical adhesion between cells.^173^ However, the current research on this type of adhesion and the materials inspired by it is often at the molecular and cellular level, which is not the same as the various macroscopic tissue adhesion mentioned above.^174^, ^175^ It is promising and exciting to mediate tissue‐specific adhesion behavior of biomedical adhesives by applying the principles of cell adhesion to the macroscopic level.

**FIGURE 7:**
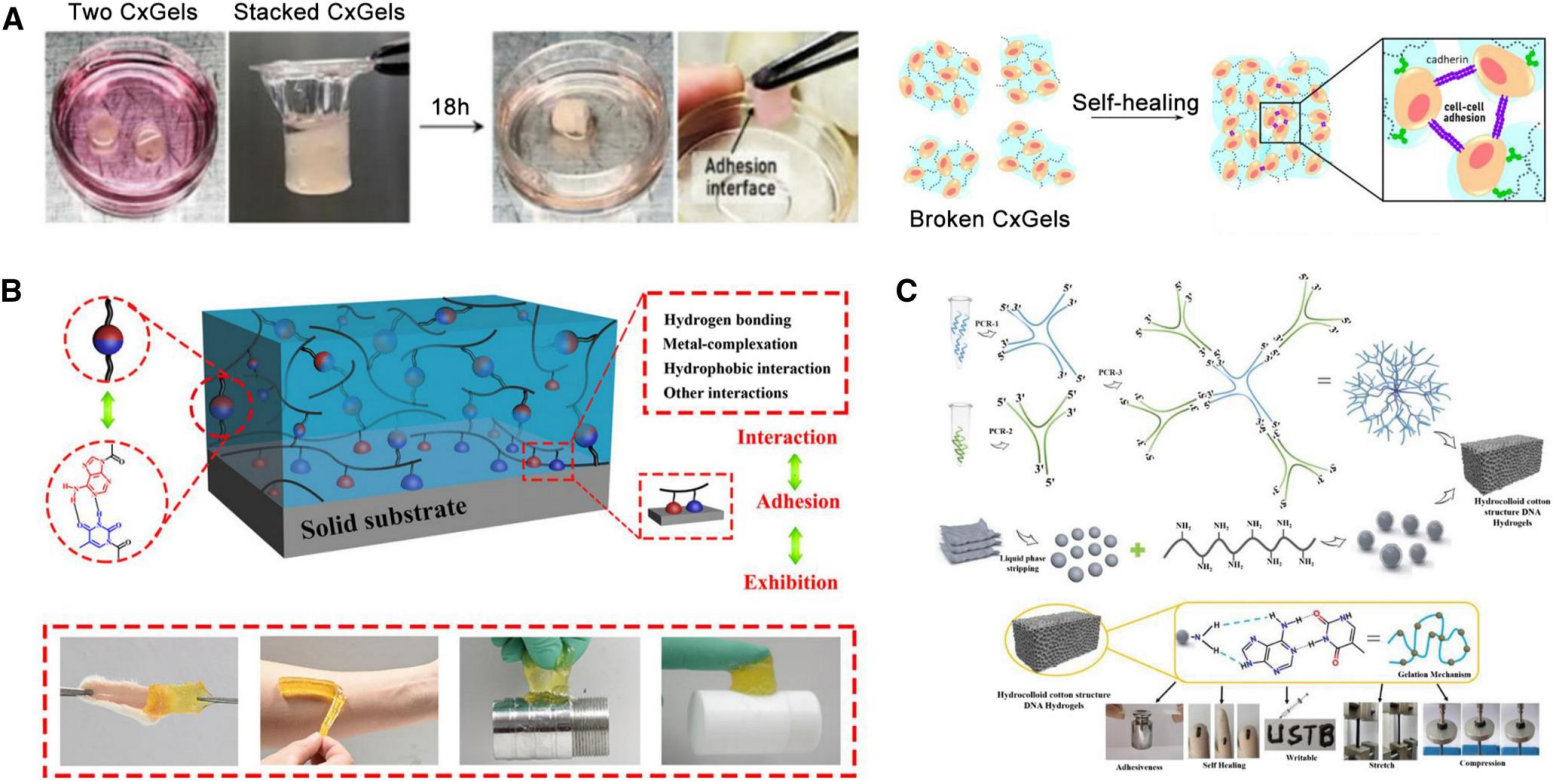
Bio‐inspired artificial hydrogels with molecular complementary pairing. (A) Materials and cells are modified to form biological tissue‐inspired hydrogels through bioorthogonal reactions and its adhesion behavior depended on cadherin‐mediated specific cell adhesion. Reproduced with permission.^180^ Copyright 2021, American Chemical Society. (B) The introduction of adenine and thymine into the hydrogel drives its adhesion behavior. Reproduced with permission.^181^ Copyright 2017, American Chemical Society. (C) The designed oligonucleotides form a dendritic‐structured linker through complementary base pairing, which is further cross‐linked with polyethylenimine‐modified black phosphorus quantum dots to form an adhesive hydrogel. Reproduced with permission.^182^ Copyright 2021, Wiley‐VCH.

Inspired by such effects, the introduction of specific cell adhesion molecules or fragments into materials is a means of promoting cell adhesion. The most common type is the introduction of adhesion molecules such as RGD, cadherin, and certain similar fragments into hydrogels.^176^, ^177^, ^178^ For example, Chakraborty et al. used the principle of supramolecular self‐assembly to construct RGD‐based hydrogels. Due to the existence of RGD fragments, the cells seeded on it were easy to adhere to the surface and connect with each other and spread.^176^ Sun et al. used synthetic biology to directly introduce fragments such as RGD to design cell adhesion proteins.^179^ Nagahama et al., took inspirations from biological tissues and designed a cadherin‐mediated “living hydrogel” system (Figure 7A).^180^ Specifically, individual modified living cells were covalently linked to the hydrogel network via bioorthogonal reactions and cells adhered to each other via cadherin‐mediated adhesion. The resulting hydrogels can form good adhesion and show self‐healing ability. The same team also verified selective adhesion properties of similar “living hydrogels” to different substrate surfaces mediated by cell adhesion.^183^

Another interesting and instructive biocomplementary pairing effect occurs in nucleobases. Nucleobases include adenine (A), thymine (T), guanine (G), cytosine (C), and uracil (U), which are important components of nucleic acids. There are pairing interactions between bases based on hydrogen bonding, including G‐C and A‐T (A‐U in RNA) (Figure 5G). This mediates pairwise binding of biological macromolecules, such as DNA and RNA.^130^ A lot of studies have applied nucleobase‐inspired adhesion to hydrogels (Figure 7B,C).^116^, ^181^, ^182^, ^184^, ^185^, ^186^ Liu et al. covalently introduced five nucleobases A, T, G, C, and U into PAAm hydrogel.^116^ All nucleobase‐containing hydrogels exhibited better adhesion than pure PAAm hydrogels. Abundant molecular recognition interactions between the hydrogels and solid surfaces, including hydrogen bonding, π‐π stacking, metal complexation, hydrophobic interaction, etc., were considered as contributing factors. Such adhesion behaviors are somehow nonspecific due to the presence of hydrophobic molecules, metal atoms, benzene rings, etc., commonly found on the substrate surface in related experiments. Other studies have used the specific hydrogen bonding pairing of A‐T and G‐C to enhance the self‐adhesion of the hydrogel network and achieve self‐healing (Figure 7B).^181^, ^184^ In addition to the introduction of nucleobase groups, single‐stranded oligonucleotides have been used as building blocks to construct dendrimer‐like DNA‐linker through hybridization, which were soon cross‐linked with a cationic polymer to form DNA‐based hydrogels (Figure 7C).^182^

### 4.3 Molecule–network interactions

The molecule–network interactions discussed here mainly include entanglement (Figure 5H) and topohesion (Figure 5I). In the mucus secreted by organisms in nature, these effects are commonly found.^26^, ^52^, ^187^, ^188^ Physically, both entanglement and topohesion can occur at an interface without any functional groups. Entanglement refers to the diffusion of polymer chains at the interface of two pre‐existing networks and physical entanglement with both networks, which results in strong adhesion. Such adhesive interfaces can be slowly separated. At the microscopic level, the polymer chains are slowly pulled out of the network at the adhesion interface during the separation process.^189^, ^190^ This type of adhesion has been likened to an unknotted suture.^26^ The topohesion theory was mainly proposed by Suo et al.^22^, ^26^, ^131^, ^132^ The initial stage of formation is the same as that of entanglement, yet topohesion arises from the formation of a third network after diffusion and entanglement.^22^, ^191^ What's more, the polymer chain diffused at the interface needs to be cross‐linked in situ to form a third network, which is entangled with the two previous networks. This usually results in a stronger adhesion than simple entanglement.^26^ It is worth mentioning that some studies have used similar principles, such as diffusing monomers at the interface and in situ cross‐linking to form networks.^192^, ^193^ Cohesion is another interesting point of view to describe the interaction of a third network between two surfaces. These correlative perspectives are expected to develop further along with the topohesion theory.^194^, ^195^

**FIGURE 8:**
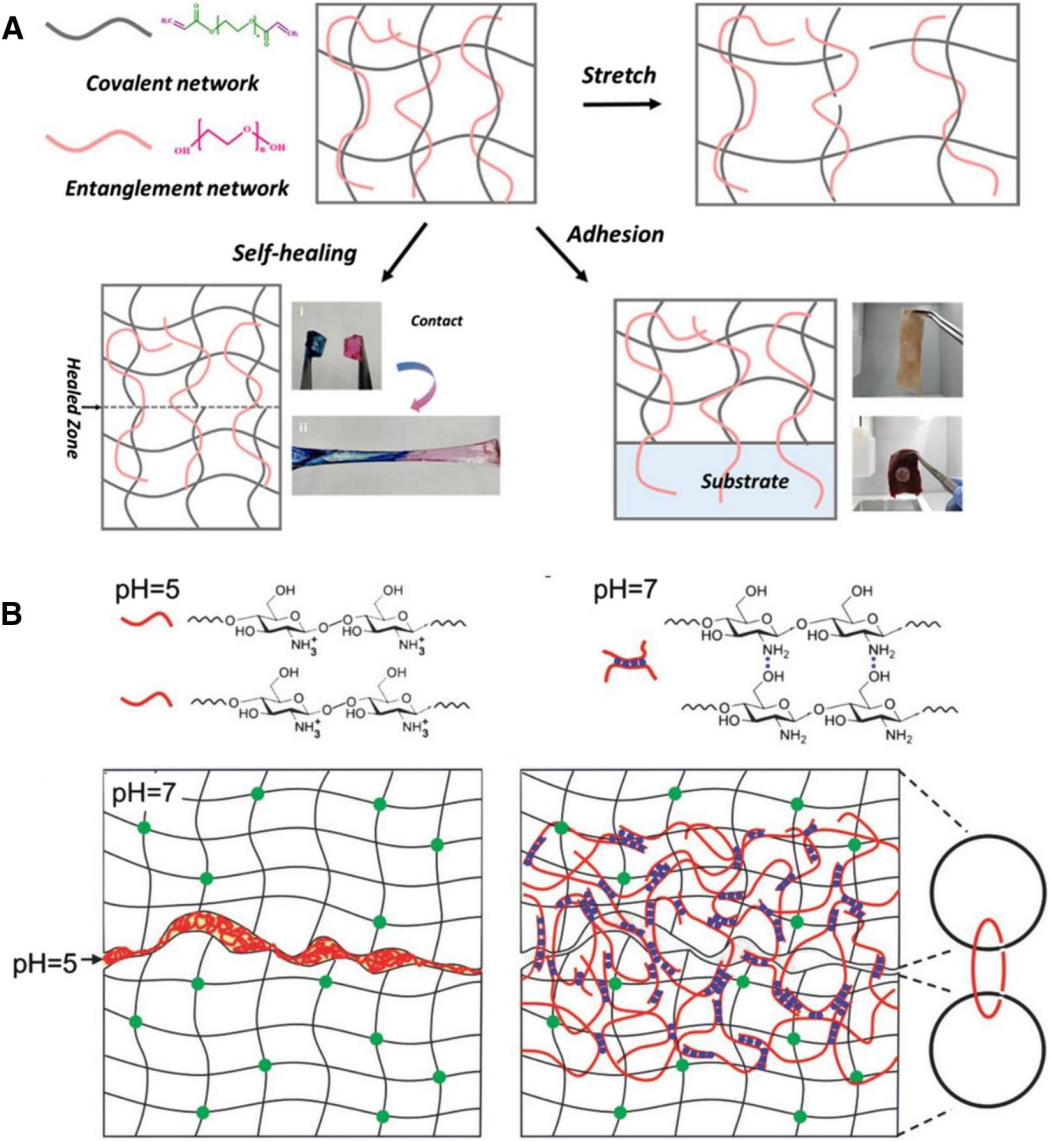
Bio‐inspired artificial hydrogels with molecule–network interactions. (A) The linear polymers are mixed in the polymer network to achieve self‐healing and adhesion properties through diffusion and entanglement. Reproduced with permission.^196^ Copyright 2019, American Chemical Society. (B) Chitosan diffuses into the network and subsequently gels in a pH‐responsive manner, forming topohesion between the networks. Reproduced with permission.^22^ Copyright 2018, Wiley‐VCH.

Similar to such an effect in nature, the entanglement strategy in artificial adhesive hydrogels is to utilize direct diffusion of, for example, macromolecules at the interface.^196^, ^197^, ^198^ For example, Chen et al. used covalently polymerized PEGDA networks and diffusible hydrophilic linear PEG molecules to form double‐network hydrogels (Figure 8A). The PEG chains in the PEGDA network can penetrate target surfaces and form entanglements, thereby generating adhesion.^196^ There are also studies that involve the use of nanomaterials to obtain hydrogels with abundant entanglements and strong adhesion.^199^ In addition, controllable entanglement can be achieved through exerting ultrasound or electric field on the polymer and tissue matrix to control adhesion.^200^, ^201^ Inspired by the pH‐triggered adhesion of mussels, Yang et al. added chitosan solution (pH = 5) at the interface of two layers of PAAm hydrogels (pH = 7). Chitosan gradually diffused and entangled to the two layers of PAAm. With the homogenization of pH, the chitosan itself gradually formed a third network via hydrogen bond cross‐linking and finally resulted in high adhesive energy (Figure 8B).^22^

### 4.4 Phase behaviors

Many bioadhesion systems are dominated by phase behaviors that involve complex phase separations, phase transitions, etc.^55^, ^58^, ^61^, ^133^, ^202^ Sandcastle worms and mussels secrete adhesives underwater. During the adhesion process, the adhesive is stable underwater without dispersion and can be applied to a substrate surface even when subjected to destructive currents.^55^ The related effect is so‐called coacervation (Figure 5J).^55^, ^133^ Coacervate is an aqueous phase rich in macromolecules, typically oppositely charged polyions. Coacervation is the phase separation of coacervate out of a dilute, equilibrium phase. The relevant molecular interactions that lead to coacervation include long‐range electrostatic forces as well as short‐range hydrogen bonding, hydrophobic interactions, etc.^57^ Coacervation makes the adhesives to not be easily dispersed in the external liquid, and thus plays an important assisted role in underwater adhesion.^55^ In addition, this process is also accompanied by solidification, that is, liquid–solid phase transition of the adhesives that makes the adhesion firm (Figure 5K).^55^, ^126^, ^203^ Another common type of phase transition related to bioadhesion is biomineralization, a process in which organisms generate solid‐phase biominerals with the help of ions through the influence of organics.^58^ Cartilage and tendon can firmly adhere to bone surfaces through a mineralized transition layer (Figure 5L).^25^, ^204^, ^205^ The inter‐binding forces provided by biomineralization between organic and inorganic matter allow the formation of hybrid materials that can be used for adhesion.^202^

**FIGURE 9:**
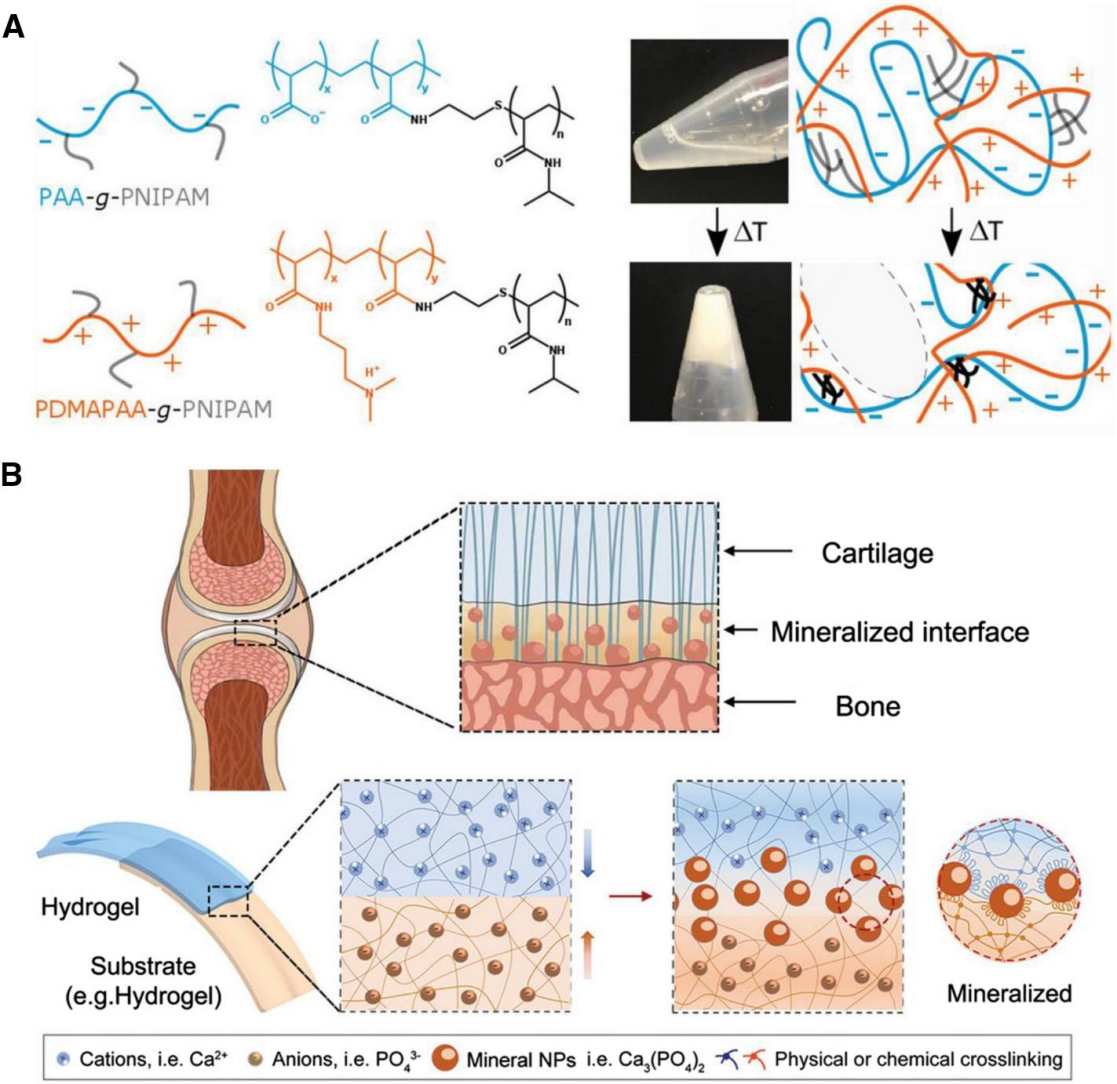
Bio‐inspired artificial hydrogels with phase behavior‐associated adhesion mechanisms. (A) Temperature‐sensitive coacervate formed by pNIPAM‐grafted oppositely charged polymers for underwater adhesion. Reproduced with permission.^134^ Copyright 2019, The Authors, published by Wiley‐VCH. (B) Cartilage‐inspired adhesion through the interaction of mineral NPs and the network. Reproduced with permission.^59^ Copyright 2022, Wiley‐VCH.

Inspired by the coacervation and solidification behaviors of sandcastle worms and mussel adhesives, various types of water‐stable, nondispersing hydrogel adhesives have been designed.^134^, ^135^, ^206^ The most common strategy is to use electrostatic forces between polyanions and polycations. Shao et al. used polyanions containing phosphate and catechol and polycations with amine groups. When the two compositions mixed with each other, a dense coacervate formed. A subsequent covalent cross‐linking process dominated by catechols resulted in firm underwater attachment.^206^ Dompé et al. grafted pNIPAM chains in polyelectrolytes to form a coacervate with thermal‐responsive phase transition ability. This made it injectable at room temperature while forming a hydrogel at body temperature, thus being favorable for underwater adhesion (Figure 9A).^134^​ In addition to conventional coacervation formed from oppositely charged molecules, Kim et al. used two polyelectrolytes of the same charge, by which the π‐cation interactions overcome electrostatic repulsion to generate an adhesive coacervate.^135^ Hydrogen bonds, hydrophobic forces, etc., have also been studied to drive coacervation.^207^, ^208^, ^209^ Besides, biomineralization also inspires studies on the adhesion of hybrid hydrogels.^59^, ^210^ Zhang et al. devised a robust and universal strategy to build stable adhesions between hydrogels and various substrates, inspired by biomineralization at the cartilage–bone interface (Figure 9B).^59^ Cation and anion pairs that can form solid minerals (e.g., calcium ions and phosphates) were introduced in the hydrogel and matrix, respectively. When the hydrogel came into contact with the matrix, ions diffused at the interface, forming mineral nanoparticles. The nanoparticles can bind to polymer chains on both sides of the surface, leading to adhesion.

At the end of the discussion in this chapter, it is worth noting that bioinspired adhesion strategies can incorporate theoretical models at multiple levels. For example, in some strategies inspired by biomineralization, inorganic particles generated at the interface bind to the networks on both sides, in a way that is similar with topohesion.^59^, ^211^ Another example is that the adhesion on a surface provided by some coacervate systems still depend on sticky groups, coagulation, etc.^134^, ^209^ Besides, some bioinspired adhesions require systematic exploration. For example, the interesting specificity of nucleobase‐dependent adhesion to various surfaces are still looking for further experiments.^116^, ^186^ Moreover, the exciting combination of adhesion theory and biotechnology and the research on adhesion of cell‐loaded “living” hydrogels have enriched the field of bio‐inspired adhesion, although much endeavor is still to be made.^179^, ^180^, ^212^

## 5 CONCLUSIONS AND PERSPECTIVES

Study on biological adhesive phenomena has come a long way, and the design of biomimetic hydrogels involves multidisciplinary efforts. For example, the study of adhesion structures and molecules in nature has promoted the generation of a series of hypotheses.^20^, ^21^, ^109^ The experimental verification and simulation help to shape the theory of adhesion.^85^, ^102^, ^213^ The combination of advanced technologies from fields such as materials science facilitate the design and construction of bio‐inspired adhesive hydrogels. In this long and extensive development process, progress always continues and discussion remains accompanied. Typical examples include the exploration on gecko toes' adhesion from different perspectives^91^, ^93^, ^94^ and the expansion of the adhesion theory with the emerging understanding on coacervation^55^, ^56^, ^57^ and topohesion,^22^, ^131^, ^132^ etc.

This article divides bio‐inspired adhesion strategies into structure‐related and molecule‐related, which is supposed to be a relatively comprehensive and systematic classification. In structure‐related adhesion, the classification revolves around the origin of the adhesion, that is, whether the adhesion arises from direct interfacial interactions, is fluid‐mediated, or does not even originate from the interface (e.g., negative pressure). These adhesion theories are still being updated as research on natural biological structure progresses. In molecule‐related adhesion, the classification mainly relies on the scale at which the force is applied with their unique characteristics—from chemical groups to molecules, networks, and phases. Theoretical models may be derived from a single effect, for example, topohesion without any sticky groups or catechol chemistry that does not involve any network interactions, both of which have been shown to result in appreciable adhesion.^26^, ^214^ However, situations in nature are often complex and some natural phenomena simultaneously involve coacervation, sticky groups, phase transitions, topohesion, etc., such as sandcastle worms and mussels. Accordingly, biomimetic strategies for creating adhesive hydrogels can also include several contributing factors.

Disciplines such as material science and engineering have played an important role in the process of converting the adhesive theories into design principles for the fabrication of bio‐inspired adhesive hydrogels. In recent years, various stimuli‐responsive smart hydrogels have shown their potential.^215^, ^216^, ^217^ For example, the temperature‐sensitive material PNIPAm has been integrated with structures inspired by geckos, tree frogs, and octopuses or chemically by the introduction of mussel‐inspired catechol groups, resulting in temperature‐controlled adhesion behavior.^117^, ^118^, ^134^, ^153^ Besides temperature control, more control strategies for the adhesion behavior of hydrogels have also been applied, such as magnetic field, electric field, light field, ultrasonic wave, etc.^197^, ^218^, ^219^ New technologies such as microfluidics, photoetching, and 3D printing can help to obtain adhesive hydrogels with finer micro–nano structures.^112^, ^119^, ^220^, ^221^, ^222^ We are confident to see that bio‐inspired adhesive hydrogels are not just blunt imitations of natural strategies, but combine the unique advantages of various advanced materials and technologies. It is believed that with the development of materials science and other fields, biomedical hydrogels that perfectly meet the requirements of safety, robustness, appropriateness, and controllability will be developed.

## AUTHOR CONTRIBUTIONS

Luoran Shang and Wenzhao Li conceived the idea; Wenzhao Li, Luoran Shang, and Xinyuan Yang wrote and revised the manuscript. Puxiang Lai assisted with the scientific discussion of the article.

## CONFLICT OF INTEREST

The authors declare that they have no known competing financial interests or personal relationships that could have appeared to influence the work reported in this paper. Luoran Shang is a member of the *Smart Medicine* editorial board.
